# Ten simple rules for carrying out and writing meta-analyses

**DOI:** 10.1371/journal.pcbi.1006922

**Published:** 2019-05-16

**Authors:** Diego A. Forero, Sandra Lopez-Leon, Yeimy González-Giraldo, Pantelis G. Bagos

**Affiliations:** 1 Laboratory of NeuroPsychiatric Genetics, Biomedical Sciences Research Group, School of Medicine, Universidad Antonio Nariño, Bogotá, Colombia; 2 PhD Program in Health Sciences, School of Medicine, Universidad Antonio Nariño, Bogotá, Colombia; 3 Novartis Pharmaceuticals Corporation, East Hanover, New Jersey, United States of America; 4 Departamento de Nutrición y Bioquímica, Facultad de Ciencias, Pontificia Universidad Javeriana, Bogotá., Colombia; 5 Department of Computer Science and Biomedical Informatics, University of Thessaly, Lamia, Greece; Dassault Systemes BIOVIA, UNITED STATES

## Introduction

In the context of evidence-based medicine, meta-analyses provide novel and useful information [1], as they are at the top of the pyramid of evidence and consolidate previous evidence published in multiple previous reports [2]. Meta-analysis is a powerful tool to cumulate and summarize the knowledge in a research field [3]. Because of the significant increase in the published scientific literature in recent years, there has also been an important growth in the number of meta-analyses for a large number of topics [4]. It has been found that meta-analyses are among the types of publications that usually receive a larger number of citations in the biomedical sciences [5,6]. The methods and standards for carrying out meta-analyses have evolved in recent years [7–9].

Although there are several published articles describing comprehensive guidelines for specific types of meta-analyses, there is still the need for an abridged article with general and updated recommendations for researchers interested in the development of meta-analyses. We present here ten simple rules for carrying out and writing meta-analyses.

### Rule 1: Specify the topic and type of the meta-analysis

Considering that a systematic review [10] is fundamental for a meta-analysis, you can use the Population, Intervention, Comparison, Outcome (PICO) model to formulate the research question. It is important to verify that there are no published meta-analyses on the specific topic in order to avoid duplication of efforts [11]. In some cases, an updated meta-analysis in a topic is needed if additional data become available. It is possible to carry out meta-analyses for multiple types of studies, such as epidemiological variables for case-control, cohort, and randomized clinical trials. As observational studies have a larger possibility of having several biases, meta-analyses of these types of designs should take that into account. In addition, there is the possibility to carry out meta-analyses for genetic association studies, gene expression studies, genome-wide association studies (GWASs), or data from animal experiments. It is advisable to preregister the systematic review protocols at the International Prospective Register of Systematic Reviews (PROSPERO; https://www.crd.york.ac.uk/Prospero) database [12]. Keep in mind that an increasing number of journals require registration prior to publication.

### Rule 2: Follow available guidelines for different types of meta-analyses

There are several available general guidelines. The first of such efforts were the Quality of Reports of Meta-analyses of Randomized Controlled Trials (QUORUM) [13] and the Meta-analysis of Observational Studies in Epidemiology (MOOSE) statements [14], but currently, the Preferred Reporting Items for Systematic reviews and Meta-analyses (PRISMA) [15] has been broadly cited and used. In addition, there have been efforts to develop specific guidelines regarding meta-analyses for clinical studies (Cochrane Handbook; https://training.cochrane.org/handbook), genetic association studies [16], genome-wide expression studies [17], GWASs [18], and animal studies [19].

### Rule 3: Establish inclusion criteria and define key variables

You should establish in advance the inclusion (such as type of study, language of publication, among others) and exclusion (such as minimal sample size, among others) criteria. Keep in mind that the current consensus advises against strict criteria concerning language or sample size. You should clearly define the variables that will be extracted from each primary article. Broad inclusion criteria increase heterogeneity between studies, and narrow inclusion criteria can make it difficult to find studies; therefore, a compromise should be found. Prospective meta-analyses, which usually are carried out by international consortia, have the advantage of the possibility of including individual-level data [20].

### Rule 4: Carry out a systematic search in different databases and extract key data

You can carry out your systematic search in several bibliographic databases, such as PubMed, Embase, The Cochrane Central Register of Controlled Trials, Scopus, Web of Science, and Google Scholar [21]. Usually, searching in several databases helps to minimize the possibility of failing to identify all published studies [22]. In some specific areas, searching in specialized databases is also worth doing (such as BIOSIS, Cumulative index to Nursing and Allied Health Literature (CINAHL), PsycINFO, Sociological Abstracts, and EconLit, among others). Moreover, in other cases, direct search for the data is also advisable (i.e., Gene Expression Omnibus [GEO] database for gene expression studies) [23]. Usually, the bibliography of review articles might help to identify additional articles and data from other types of documents (such as theses or conference proceedings) that might be included in your meta-analysis. The Web of Science database can be used to identify publications that have cited key articles. Adequate extraction and recording of key data from primary articles are fundamental for carrying out a meta-analysis. Quality assessment of the included studies is also an important issue; it can be used for determining inclusion criteria, sensitivity analysis, or differential weighting of the studies. For example the Jadad scale [24] is frequently used for randomized clinical trials, the Newcastle–Ottawa scale [25] for nonrandomized studies, and QUADAS-2 for the Quality Assessment of Diagnostic Accuracy Studies [26]. It is recommended that these steps be carried out by two researchers in parallel and that discrepancies be resolved by consensus. Nevertheless, the reader must be aware that quality assessment has been criticized, especially when it reduces the studies to a single “quality” score [27,28]. In any case, it is important to avoid the confusion of using guidelines for the reporting of primary studies as scales for the assessment of the quality of included articles [29,30].

### Rule 5: Contact authors of primary articles to ask for missing data

It is common that key data are not available in the main text or supplementary files of primary articles [31], leading to the need to contact the authors to ask for missing data. However, the rate of response from authors is lower than expected. There are multiple standards that promote the availability of primary data in published articles, such as the minimum information about a microarray experiment (MIAME) [32] and the STrengthening the REporting of Genetic Association Studies (STREGA) [33]. In some areas, such as genetics, in which it was shown that it is possible to identify an individual using the aggregated statistics from a particular study [34], strict criteria are imposed for data sharing, and specialized permissions might be needed.

### Rule 6: Select the best statistical models for your question

For cases in which there is enough primary data of adequate quality for a quantitative summary, there is the option to carry out a meta-analysis. The potential analyst must be warned that in many cases the data are reported in noncompatible forms, so one must be ready to perform various types of transformations. Thankfully, there are methods available for extracting and transforming data regarding continuous variables [35–37], 2 × 2 tables [38,39], or survival data [40]. Frequently, meta-analyses are based on fixed-effects or random-effects statistical models [20]. In addition, models based on combining ranks or *p*-values are also available and can be used in specific cases [41–44]. For more complex data, multivariate methods for meta-analysis have been proposed [45,46]. Additional statistical examinations involve sensitivity analyses, metaregressions, subgroup analyses, and calculation of heterogeneity metrics, such as Q or I^2^ [20]. It is fundamental to assess and, if present, explain the possible sources of heterogeneity. Although random-effects models are suitable for cases of between-studies heterogeneity, the sources of between-studies variation should be identified, and their impact on effect size should be quantified using statistical tests, such as subgroup analyses or metaregression. Publication bias is an important aspect to consider [47], since in many cases negative findings have less probability of being published. Other types of bias, such as the so-called “Proteus phenomenon” [48] or “winner’s curse” [49], are common in some scientific fields, such as genetics, and the approach of cumulative meta-analysis is suggested in order to identify them.

### Rule 7: Use available software to carry metastatistics

There are several very user-friendly and freely available programs for carrying out meta-analyses [43,44], either within the framework of a statistical package such as Stata or R or as stand-alone applications. Stata and R [50–52] have dozens of routines, mostly user written, that can handle most meta-analysis tasks, even complex analyses such as network meta-analysis and meta-analyses of GWASs and gene expression studies (https://cran.r-project.org/web/views/MetaAnalysis.html; https://www.stata.com/support/faqs/statistics/meta-analysis). There are also stand-alone packages that can be useful for general applications or for specific areas, such as OpenMetaAnalyst [53], NetworkAnalyst [54], JASP [55], MetaGenyo [56], Cochrane RevMan (https://community.cochrane.org/help/tools-and-software/revman-5), EpiSheet (krothman.org/episheet.xls), GWAR [57], GWAMA [58], and METAL [59]. Some of these programs are web services or stand-alone software. In some cases, certain programs can present issues when they are run because of their dependency on other packages.

### Rule 8: The records and study report must be complete and transparent

Following published guidelines for meta-analyses guarantees that the manuscript will describe the different steps and methods used, facilitating their transparency and replicability [15]. Data such as search and inclusion criteria, numbers of abstracts screened, and included studies are quite useful, in addition to details of meta-analytical strategies used. An assessment of quality of included studies is also useful [60]. A spreadsheet can be constructed in which every step in the selection criteria is recorded; this will be helpful to construct flow charts. In this context, a flow diagram describing the progression between the different steps is quite useful and might enhance the quality of the meta-analysis [61]. Records will be also useful if, in the future, the meta-analysis needs to be updated. Stating the limitations of the analysis is also important [62].

### Rule 9: Provide enough data in your manuscript

A table with complete information about included studies (such as author, year, details of included subjects, DOIs, or PubMed IDs, among others) is quite useful in an article reporting a meta-analysis; it can be included in the main text of the manuscript or as a supplementary file. Software used for carrying out meta-analyses and to generate key graphs, such as forest plots, should be referenced. Summary effect measures, such as a pooled odds ratios or the counts used to generate them, should be always reported, including confidence intervals. It is also possible to generate figures with information from multiple forest plots [63]. In the case of positive findings, plots from sensitivity analyses are quite informative. In more-complex analyses, it is advisable to include in the supplementary files the scripts used to generate the results [64].

### Rule 10: Provide context for your findings and suggest future directions

The Discussion section is an important scientific component in a manuscript describing a meta-analysis, as the authors should discuss their current findings in the context of the available scientific literature and existing knowledge [65]. Authors can discuss possible reasons for the positive or negative results of their meta-analysis, provide an interpretation of findings based on available biological or epidemiological evidence, and comment on particular features of individual studies or experimental designs used [66]. As meta-analyses are usually synthesizing the existing evidence from multiple primary studies, which commonly took years and large amounts of funding, authors can recommend key suggestions for conducting and/or reporting future primary studies [67].

As open science is becoming more important around the globe [68,69], adherence to published standards, in addition to the evolution of methods for different meta-analytical applications, will be even more important to carry out meta-analyses of high quality and impact.
